# CHA_2_DS_2_-VASc score for in-hospital recurrence risk stratification in patients with myocardial infarction

**DOI:** 10.3389/fcvm.2022.925932

**Published:** 2022-12-01

**Authors:** Hui Pang, Xu Zhu, Iokfai Cheang, Haifeng Zhang, Yanli Zhou, Shengen Liao, Xinli Li

**Affiliations:** ^1^Department of Cardiology, The First Affiliated Hospital of Nanjing Medical University, Nanjing, Jiangsu, China; ^2^Department of Cardiology, The Affiliated Suzhou Hospital of Nanjing Medical University, Suzhou, Jiangsu, China

**Keywords:** CHA_2_DS_2_-VASc, myocardial infarction, recurrence, sex differences, risk stratification

## Abstract

**Background:**

Using the CHA_2_DS_2_-VASc score to recognize the risk of stroke in patients with atrial fibrillation has been well-established. However, few studies have assessed whether the CHA_2_DS_2_-VASc score has a similar predictive value in recurrence after myocardial infarction (MI).

**Methods:**

We conducted a retrospective observational cohort study of adult inpatients with MI. The CHA_2_DS_2_-VASc and modified CHA_2_DS_2_-VASc (MCHA_2_DS_2_-VASc) scores of all patients were calculated. The associations of both scores with recurrent MI were analyzed.

**Results:**

A total of 6,700 patients with MI (60.0 ± 11.1 years, 77.2% men) were enrolled, and 759 (11.3%) presented a definite recurrence during hospitalization. After multivariable adjustment by logistic regression in patients with MI, the CHA_2_DS_2_-VASc and MCHA_2_DS_2_-VASc scores were independently associated with recurrence. The MCHA_2_DS_2_-VASc score showed a better predictive value for risk of recurrence than that of CHA_2_DS_2_-VASc in overall [area under the receiver operating characteristic curve (AUC) 0.757 vs. 0.676] or male patients (AUC 0.759 vs. 0.708). MCHA_2_DS_2_-VASc was superior to CHA_2_DS_2_-VASc for identifying “truly high-risk” patients with MI, regardless of overall patients or sex-specific subgroups. The two scores had a similar focus on the identification of “low-risk” patients in overall or women, but not in men.

**Conclusion:**

The CHA_2_DS_2_-VASc and MCHA_2_DS_2_-VASc scores for predicting recurrence are validated in patients with MI. However, MCHA_2_DS_2_-VASc could be more helpful to secondary prevention than CHA_2_DS_2_-VASc after MI, especially in men. The superiority of MCHA_2_DS_2_-VASc compared with CHA_2_DS_2_-VASc in women is just more discriminatory for “truly high-risk” patients.

## Introduction

In recent years, morbidity and mortality from coronary heart disease (CHD) have been declining in most developed countries but rising in other low-and middle-income countries. Globally, death and disability rates from CHD in patients presenting with myocardial infarction (MI) remain high when the quality of medical and health services is improved remarkably (1). Patients with recurrent MI often have a poor prognosis, largely due to a reduction in cardiac pump activity and malignant arrhythmias, and even trigger sudden cardiac death (2, 3). Better management *via* risk stratification is imperative for short- and long-term secondary prevention of MI. A number of clinically applicable cardiovascular risk-stratification schemes have been proven as efficient tools for risk stratification and therapeutic decision-making, such as Thrombolysis in Myocardial Infarction (TIMI) risk score, which is based on nine clinical characteristics (4).

Major international atrial fibrillation (AF) guidelines recommend estimating stroke risk in patients with AF based on the CHA_2_DS_2_-VASc score, which summarizes common stroke risk factors. CHA_2_DS_2_-VASc score performs just modestly in predicting high-risk patients, and its advantage is better at identifying “truly low-risk” patients with AF who develop stroke and thromboembolism (5).

Many clinical stroke risk factors (e.g., obstructive sleep apnea, left atrium dilatation, and renal impairment) (6–8) as well as some biomarkers (e.g., troponin, natriuretic peptides, and von Willebrand factor) (9, 10) are closely related to the stroke risk, but they do not improve the predictive value of CHA_2_DS_2_-VASc score. Moreover, patients with MI seem to be at increased risk for recurrent major adverse cardiovascular events (MACEs), owing to their clinical characteristics, comorbidities, and biomarkers, such as elderly (especially older women) (11), diabetes mellitus (DM), heart failure (HF), renal dysfunction (12), and interleukin-1beta (13). Coincidentally, there are some concordant factors between stroke and recurrent MI, which are two different disorders. Therefore, this study was conducted to evaluate whether the CHA_2_DS_2_-VASc score could assess the risk of recurrence in patients with MI, presented overall and stratified by sex.

## Materials and methods

### Study population

A retrospective observational cohort of 6,700 adult inpatients in the First Affiliated Hospital of Nanjing Medical University, Affiliated Hospital of Xuzhou Medical University, and Xuzhou Central Hospital between January 2019 and December 2021 was evaluated. This study was conducted on inpatients with types 1–3 MI but not types 4–5 procedure-related MI, which occurred more than 28 days at enrollment (2). The history of MI was ascertained based on self-reporting or medical records. Patients with recurrent MI were enrolled who underwent acute myocardial infarction (AMI) during hospitalization. All patients had an MI attack for the first time during enrollment. Subjects with recurrent MI diagnosed prior to the enrollment were excluded. In addition, the study excluded patients who had congenital cardiovascular diseases, idiopathic cardiomyopathy, rheumatic mitral stenosis, mechanical or bioprosthetic heart valve, or mitral valve repair, malignant tumor, liver or kidney failure, major bleeding, and immune diseases. This study was approved by the Ethics Committee of the First Affiliated Hospital of Nanjing Medical University (2022-SR-062) under a waiver of informed consent, in accordance with ethical guidelines set up by the World Medical Association (The Declaration of Helsinki).

### Data collection

Investigators collected epidemiological, demographic, clinical, and outcome data from electronic medical records, such as each patient’s age, sex, and history of hypertension, DM, HF, thromboembolism, dyslipidemia, and AF. All information was recorded on a computerized database using a standardized electronic data collection form, which serves as the data source of this study.

### Definitions and outcome measures

Each patient’s CHA_2_DS_2_-VASc score ranging from 0 to 9 was calculated [patients were given 1 point for HF, hypertension, age 65–74 years, DM, vascular disease, and female sex and 2 points for age ≥ 75 years and prior stroke/transient ischemic attack (TIA)/embolus] using baseline characteristics. Modified CHA_2_DS_2_-VASc (MCHA_2_DS_2_-VASc) to give 1 point for age ≥ 75 years, DM, and dyslipidemia, 2 points for prior stroke/TIA/embolus and male sex, 3 points for hypertension, and 5 points for HF, ranged from 0 to 15. The optimal cutoff point of CHA_2_DS_2_-VASc was 3 and MCHA_2_DS_2_-VASc was 8 for predicting the incidence of recurrent MI, which was determined by receiver operating characteristic (ROC) curve, area under the curve (AUC) analysis, and Youden index. Patients were subsequently categorized into the low-risk group (0–2) and high-risk group (3–9) according to the CHA_2_DS_2_-VASc and low-risk group (0–7) and high-risk group (8–15) according to the MCHA_2_DS_2_-VASc.

The primary outcome of this study was the occurrence of AMI in hospitals. The definition of AMI based on the Fourth Universal Definition of Myocardial Infarction (2018) is as follows: a clinical (or pathological) event in the setting of evidence of acute myocardial ischemia (ischemic symptoms, ischemic electrocardiographic changes, coronary artery intervention, new wall motion abnormalities, or fixed defect on radionuclide scanning) in which there is the presence of acute myocardial cell death detected by abnormal cardiac biomarkers (14).

### Statistical analysis

Baseline characteristics of patients were described using means and standard deviations (SDs) for normally distributed continuous data and numbers and percentages for categorical data. These characteristics were compared using Student’s *t*-tests, chi-square tests, or Kruskal–Wallis tests, as appropriate. The correlations between CHA_2_DS_2_-VASc and recurrence rate and MCHA_2_DS_2_-VASc and recurrence rate were evaluated using the test of Spearman’s rank-correlation coefficient. To identify the independent predictors of in-hospital recurrent MI, a multivariate logistic regression model was performed using the following variables: age, sex, hypertension, DM, HF, thromboembolism, dyslipidemia, and AF. The results of logistic regression analysis were reported as an odds ratio (OR) with a 95% confidence interval (CI). The predictive value of CHA_2_DS_2_-VASc and MCHA_2_DS_2_-VASc with regard to recurrence was assessed using AUC in the presentation of the ROC curve. The AUC used to quantify the discriminatory capacity of the two scores for recurrence is defined as excellent (0.9–1), good (0.8–0.89), fair (0.7–0.79), poor (0.6–0.69), or fail/no discriminatory capacity (0.5–0.59) (15). Statistical significance was accepted for two-sided *p*-values < 0.05. The statistical analyses were performed using SPSS version 22.0.

## Results

### Baseline characteristics

In this study, 1,530 (22.8%) women and 5,170 (77.2%) men were included (Table 1). A total of 759 (11.3%) patients experienced a recurrent MI. Age at entry ranged from 21 to 94 years (mean age 60 years). Patients with recurrent MI were older and had higher CHA_2_DS_2_-VASc and MCHA_2_DS_2_-VASc scores than those without such disorders. In the two groups, most recurrent patients were male (81.6 vs. 76.6%). Compared with the patients without recurrence, recurrent patients were more likely to have the comorbidities of hypertension, DM, HF, thromboembolism, dyslipidemia, and AF. Most recurrent patients were ≥65 years of age, whereas those aged ≤ 64 years were responsible for the majority of patients without recurrence.

**TABLE 1:** Baseline characteristics of myocardial infarction patients with or without recurrence.

	Total (*n* = 6,700)	Recurrence		*P*-value
			
		No (*n* = 5,941)	Yes (*n* = 759)	
Age at baseline, mean (SD), years	60.0 (11.1)	59.7 (11.0)	62.9 (11.8)	< 0.001
Components of the CHA_2_DS_2_-VASc score, *n* (%)				
Age group, years				< 0.001
≤64	4216 (62.9)	3824 (64.4)	392 (51.6)	
65–74	1811 (27.0)	1576 (26.5)	235 (31.0)	
≥75	673 (10.0)	541 (9.1)	132 (17.4)	
Female	1530 (22.8)	1390 (23.4)	140 (18.4)	0.002
Hypertension	2964 (44.2)	2442 (68.8)	522 (41.1)	< 0.001
Diabetes mellitus	1904 (28.4)	1596 (26.9)	308 (40.6)	< 0.001
Heart failure	4060 (60.6)	3397 (57.2)	663 (87.4)	< 0.001
NYHA class				< 0.001
I	1420 (21.2)	1115 (18.8)	305 (40.2)	
II	1719 (25.7)	1493 (25.1)	226 (29.8)	
III	640 (9.6)	562 (9.5)	78 (10.3)	
IV	281 (4.2)	227 (3.8)	54 (7.1)	
Thromboembolism	520 (7.8)	400 (6.7)	120 (15.8)	< 0.001
Stroke/transient ischemic attack	458 (6.8)	349 (5.9)	109 (14.4)	< 0.001
Comorbidities, *n* (%)				
Dyslipidemia	2845 (42.5)	2489 (41.9)	356 (46.9)	0.009
Atrial fibrillation	341 (5.1)	283 (4.8)	58 (7.6)	0.001
CHA_2_DS_2_-VASc score, mean (SD)	3.2 (1.6)	3.1 (1.5)	4.1 (1.7)	< 0.001
MCHA_2_DS_2_-VASc score, mean (SD)	6.9 (3.2)	6.5 (3.1)	9.4 (2.7)	< 0.001

NYHA, New York Heart Association; SD, standard deviation.

### Patient characteristics and recurrent myocardial infarction

The independent predictors of recurrence analyzed by logistic regression are reported in Tables 2, 3. After multivariable adjustment, older age, male sex, hypertension, DM, HF, thromboembolism, dyslipidemia, and CHA_2_DS_2_-VASc and MCHA_2_DS_2_-VASc scores were strongly independently associated with recurrent MI. Contrary to the male sex, the female sex as a stroke-related factor in AF was not considered to be an independent recurrent MI risk factor by multivariable analysis.

**TABLE 2:** Multivariate regression analysis investigating independent predictors of recurrent myocardial infarction.

	Coefficient	S.E.	Wald	OR	95% CI	*P*-value
Age	0.011	0.004	7.343	1.011	1.003–1.018	0.007
Male	0.803	0.106	57.243	2.232	1.813–2.749	< 0.001
Hypertension	1.173	0.091	167.915	3.231	2.706–3.859	< 0.001
Diabetes mellitus	0.290	0.086	11.530	1.337	1.131–1.581	0.001
Heart failure	1.603	0.114	196.516	4.968	3.970–6.216	< 0.001
Thromboembolism	0.589	0.121	23.639	1.802	1.421–2.285	< 0.001
Dyslipidemia	0.243	0.083	8.699	1.276	1.085–1.500	0.003

The adjusted model was adjusted for age, sex, hypertension, diabetes mellitus, heart failure, thromboembolism, dyslipidemia, and atrial fibrillation.

CI, confidence interval; OR, odds ratio; SE, standard error.

**TABLE 3:** CHA_2_DS_2_-VASc and MCHA_2_DS_2_-VASc scores in predicting recurrence following myocardial infarction.

	Coefficient	S.E.	Wald	OR	95% CI	*P*-value
**CHA_2_DS_2_-VASc score**						
Model 1	0.388	0.023	278.743	1.473	1.408–1.542	<0.001
Model 2	0.624	0.033	352.294	1.867	1.749–1.993	<0.001
Model 3	0.258	0.035	54.048	1.295	1.209–1.387	<0.001
**MCHA_2_DS_2_-VASc score**						
Model 1	0.342	0.016	476.346	1.408	1.365–1.452	<0.001
Model 2	0.335	0.016	421.329	1.398	1.354–1.443	<0.001
Model 3	0.342	0.016	476.346	1.408	1.365–1.452	<0.001

Model 1: unadjusted. Model 2: adjusted for age and sex. Model 3: adjusted for age, sex, hypertension, diabetes mellitus, heart failure, thromboembolism, dyslipidemia, and atrial fibrillation. CI, confidence interval; OR, odds ratio; SE, standard error.

### Relationship between CHA_2_DS_2_-VASc, MCHA_2_DS_2_-VASc, and recurrent myocardial infarction

**FIGURE 1:**
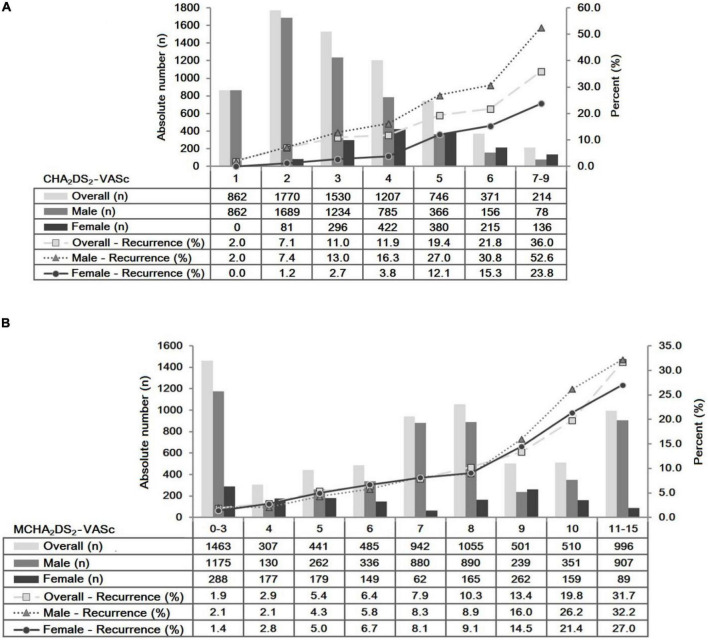
Frequency distribution and incidence of recurrent myocardial infarction for congestive heart failure, hypertension, age 65–74 years, diabetes mellitus, vascular disease, female sex, age ≥ 75 years, and prior stroke/transient ischemic attack/embolus [CHA_2_DS_2_-VASc **(A)**] and age ≥ 75 years, diabetes mellitus, dyslipidemia, prior stroke/transient ischemic attack/embolus, hypertension, male sex, and congestive heart failure [MCHA_2_DS_2_-VASc **(B)**] scores’ strata, presented overall, male sex, and female sex.

The frequency distribution of the CHA_2_DS_2_-VASc and MCHA_2_DS_2_-VASc scores, and the incidence of recurrent MI across the CHA_2_DS_2_-VASc and MCHA_2_DS_2_-VASc scores in the study cohort, stratified by sex, are shown in Figure 1. The overall incidence of recurrent MI increased from 2.0 to 33.3% when the CHA_2_DS_2_-VASc score increased from 1 to 9 (Figure 1A). In addition, patients confer an overall increased risk of recurrent MI from 1.4 to 61.5%, while the MCHA_2_DS_2_-VASc score increased from 0 to 15 (Figure 1B). The positive correlations between CHA_2_DS_2_-VASc and recurrence rate and MCHA_2_DS_2_-VASc and recurrence rate were observed, and the Spearman correlation coefficients were 0.198 (*p* < 0.001) and 0.283 (*p* < 0.001) separately. Further analyses revealed that the risk for recurrent MI incidence positively correlated with the CHA_2_DS_2_-VASc (Spearman correlation coefficient for men = 0.241 and women = 0.235) and MCHA_2_DS_2_-VASc (Spearman correlation coefficient for men = 0.294 and women = 0.237) among both women and men.

### Receiver operating characteristic curves for CHA_2_DS_2_-VASc and MCHA_2_DS_2_-VASc in predicting recurrent myocardial infarction

**FIGURE 2:**
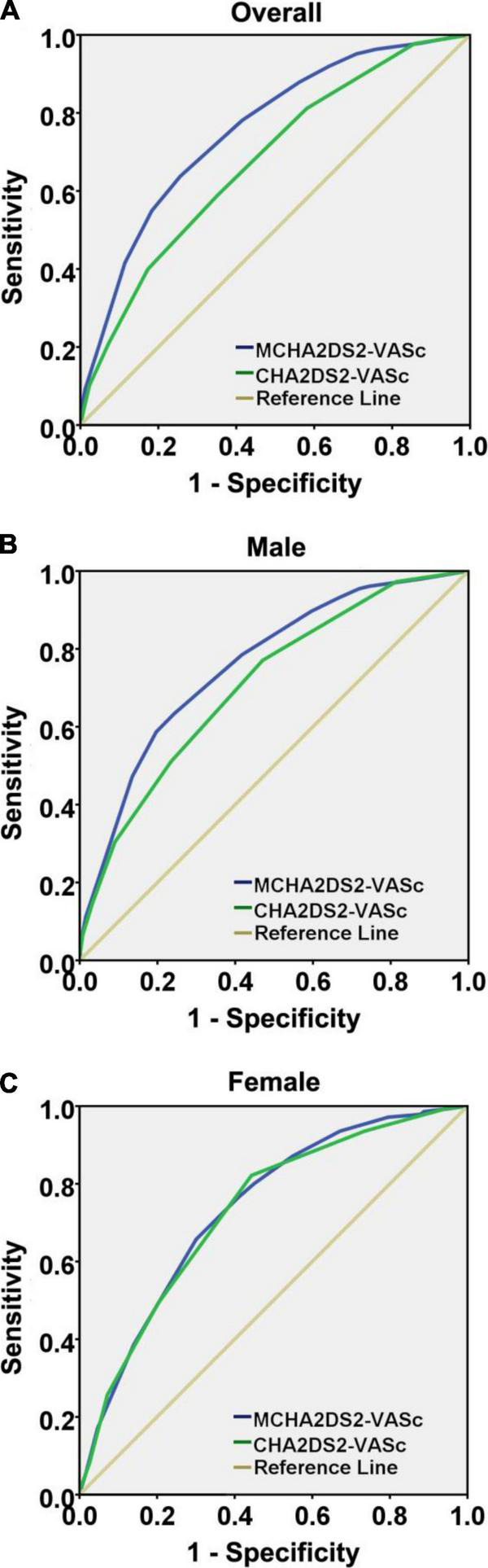
Receiver operating characteristic curve analysis comparing CHA_2_DS_2_-VASc and MCHA_2_DS_2_-VASc scores’ ability to predict recurrent myocardial infarction, presented overall **(A)**, male sex **(B)**, and female sex **(C)**.

The ROC curves of CHA_2_DS_2_-VASc and MCHA_2_DS_2_-VASc scores were analyzed (Figure 2 and Table 4). The predictive value of MCHA_2_DS_2_-VASc for recurrence was fair, being 0.757 (0.739–0.774) with a cutoff value of 8, but CHA_2_DS_2_-VASc had just a poor effect on prediction, with the AUC of 0.676 (0.657–0.696) and a cutoff value of 3 (Figure 2A). Thus, for the secondary prevention of MI, MCHA_2_DS_2_-VASc was recommended to take into consideration the recurrence risk assessment because it significantly improved the predictive value compared with the CHA_2_DS_2_-VASc (*Z* = 6.02, *p* < 0.001).

**TABLE 4:** Predictive ability, sensitivity, and specificity of CHA_2_DS_2_-VASc and MCHA_2_DS_2_-VASc scores for recurrent myocardial infarction development, presented overall, and stratified by sex.

	AUC	S.E.	*P*-value	95% CI	Sensitivity (%)	Specificity (%)	Cut off point
**CHA_2_DS_2_-VASc**							
Overall	0.676	0.010	<0.001	0.657–0.696	58.9	64.8	3
Male	0.708	0.011	<0.001	0.687–0.730	77.1	52.9	2
Female	0.730	0.021	<0.001	0.688–0.771	82.1	55.7	4
**MCHA_2_DS_2_-VASc**							
Overall	0.757	0.009	<0.001	0.739–0.774	63.8	74.4	8
Male	0.759	0.010	<0.001	0.739–0.779	63.3	75.7	8
Female	0.736	0.020	<0.001	0.696–0.776	65.7	69.9	8

AUC, area under the ROC curve; CI, confidence interval; SE, standard error.

The AUCs of CHA_2_DS_2_-VASc and MCHA_2_DS_2_-VASc scores in predicting recurrent MI in male patients were 0.708 (0.687–0.730) and 0.759 (0.739–0.779), respectively. Both the two scores had a fair predictive value for recurrence, but the two predictors showed significant differences in risk assessment (*Z* = 3.43, *p* < 0.001) (Figure 2B). However, the superiority of the MCHA_2_DS_2_-VASc score among women yielded inconsistent findings. The AUCs of CHA_2_DS_2_-VASc and MCHA_2_DS_2_-VASc scores were 0.730 (0.688–0.771) and 0.736 (0.696–0.776), respectively. The predictive abilities of the two scores were both fair, and there was no significant difference between them (*Z* = 0.21, *p* = 0.836) (Figure 2C).

### CHA_2_DS_2_-VASc, MCHA_2_DS_2_-VASc, and recurrent myocardial infarction risk stratification

Recurrent MI risk differentiation among patients presented overall and stratified by sex was classified as low risk and high risk, based on CHA_2_DS_2_-VASc and MCHA_2_DS_2_-VASc scores (Table 5). The overall patients classified as low-risk using CHA_2_DS_2_-VASc (score < 3) or MCHA_2_DS_2_-VASc (score < 8) had similarly low recurrence rates (5.4 vs. 4.6%). There was also no evidence of a statistical difference in the recurrence rates among those in women (MCHA_2_DS_2_-VASc < 8 vs. CHA_2_DS_2_-VASc < 4) identified as low risk. High-risk MCHA_2_DS_2_-VASc associated with a higher incidence of recurrent MI as compared with high-risk CHA_2_DS_2_-VASc was found in overall (19.4 vs. 15.1%), male (20.4 vs. 14.0%), and female (15.9 vs. 11.4%) patients.

**TABLE 5:** Comparison of CHA_2_DS_2_-VASc and MCHA_2_DS_2_-VASc scores for incidence of recurrent myocardial infarction, presented overall, and stratified by sex.

	Case (*n*)	Recurrence rate (%)	*P*-value
**Overall**			
Low-risk			0.116
CHA_2_DS_2_-VASc<3	2,632	143 (5.4)	
MCHA_2_DS_2_-VASc < 8	3,638	166 (4.6)	
High-risk			< 0.001
CHA_2_DS_2_-VASc ≥ 3	4,068	616 (15.1)	
MCHA_2_DS_2_-VASc ≥ 8	3,062	593 (19.4)	
**Male**			
Low-risk			< 0.001
CHA_2_DS_2_-VASc < 2	862	17 (2.0)	
MCHA_2_DS_2_-VASc < 8	2,783	133 (4.8)	
High-risk			< 0.001
CHA_2_DS_2_-VASc ≥ 2	4,308	602 (14.0)	
MCHA_2_DS_2_-VASc ≥ 8	2,387	486 (20.4)	
**Female**			
Low-risk			0.189
CHA_2_DS_2_-VASc < 4	377	9 (2.4)	
MCHA_2_DS_2_-VASc < 8	855	33 (3.9)	
High-risk			0.006
CHA_2_DS_2_-VASc ≥ 4	1,153	131 (11.4)	
MCHA_2_DS_2_-VASc ≥ 8	675	107 (15.9)	

## Discussion

This study, based on a large cohort of patients with MI admitted with a duration longer than 28 days, has the following three main findings: (i) MCHA_2_DS_2_-VASc score is a fair predictor of recurrent MI, but only poor predictive accuracy of CHA_2_DS_2_-VASc score is available, (ii) MCHA_2_DS_2_-VASc score is significantly superior to CHA_2_DS_2_-VASc score in predicting male patients who develop recurrent MI, although the predictive value of the two scores is fair in both men and women, and (iii) MCHA_2_DS_2_-VASc score is better at identifying high-risk patients and is as good as CHA_2_DS_2_-VASc score in identifying patients at low-risk of recurrence among overall and female patients.

It has been reported that the CHA_2_DS_2_-VASc score is useful to predict many different diseases. In patients with CAD and sinus rhythm, CHA_2_DS_2_-VASc exhibited moderate accuracy in predicting the risk of stroke or TIA in the period following an episode of worsening HF and reduced ejection (15). In acute coronary syndrome patients treated with aspirin and clopidogrel following PCI, CHA_2_DS_2_-VASc (AUC = 0.59) was able to predict platelet reactivity (16). Gunduz et al. found the predictive value of CHA_2_DS_2_-VASc (AUC = 0.89) for mortality, intensive care unit (ICU) hospitalization, and length of stay in the ICU among COVID-19 patients (17). Similarly, another study also reported that CHA_2_DS_2_-VASc (AUC = 0.794) was significantly associated with all-cause mortality in COVID-19 patients (18).

A large number of people do not survive their first MI event, and if they do survive, their rate of adverse cardiovascular events, hospitalization, and mortality is greater than for the non-MI population. A risk stratification tool must be considered for each patient with MI in order to identify patients at high risk for disease progression. Notably, we found that many of the risk factors for incident MI recurrence were also risk factors for AF-related complications. Thus, we developed and tested the CHA_2_DS_2_-VASc score to predict the risk for recurrence after MI.

A total of 6,700 patients with MI were included in our analysis. Baseline characteristics showed significant differences between patients with or without recurrence, such as older age, female sex, hypertension, DM, HF, thromboembolism, dyslipidemia, and AF. Moreover, patients with recurrence had higher CHA_2_DS_2_-VASc scores. A positive linear relationship in both sexes of elevated CHA_2_DS_2_-VASc with recurrent events was found. After adjustment for baseline risk, higher CHA_2_DS_2_-VASc was independently associated with higher recurrent event rates. Every 1-SD increase in CHA_2_DS_2_-VASc was associated with a 29.5% increased risk. The AUC of CHA_2_DS_2_-VASc in predicting recurrence was 0.676 in overall, 0.708 in men, and 0.730 in women.

Despite being a predictor of recurrent MI, the CHA_2_DS_2_-VASc score has just poor accuracy. Thus, we analyzed risk factors for recurrence to improve model prediction. Our data indicated that older age, male sex, hypertension, DM, HF, thromboembolism, and dyslipidemia were independently significantly associated with a higher risk of recurrence. These risk factors were summarized in the clinical risk factor-based MCHA_2_DS_2_-VASc score. There was a positive linear relationship between MCHA_2_DS_2_-VASc and recurrence rate. Our observation of associations in both crude and comorbidity-adjusted analyses suggested that MCHA_2_DS_2_-VASc acted independently of other risk factors for recurrent events. Every 1-SD increase in MCHA_2_DS_2_-VASc was associated with a 40.8% increased risk. MCHA_2_DS_2_-VASc was able to predict recurrence with an AUC of 0.757. It was noteworthy that MCHA_2_DS_2_-VASc was possibly better than CHA_2_DS_2_-VASc in identifying patients with MI with a risk of recurrence.

Heart failure is a common complication in patients with AMI, ranging from 15 to 35% of cases. Patients with HF exist in a hypercoagulable state and are at increased risk for thromboembolic events, even when in sinus rhythm (19). The presence of symptoms of HF and/or left ventricular systolic dysfunction identifies a population of MI survivors at high risk for death, reinfarction, and worsening HF (20). Reduced left ventricular ejection fraction still remains the most powerful independent predictor of sudden arrhythmic death in patients with MI. Current direct evidence noted that prior MI in heart failure with preserved ejection fraction was associated with a 31-fold higher risk of cardiovascular death in the first 30 days and persistently elevated rates of HF hospitalization (21). In view of the above published clinical trials and our findings, it seems robust to give 5 points for HF as a component of the MCHA_2_DS_2_-VASc score.

Other risk factors involved in the MCHA_2_DS_2_-VASc score, such as older age, DM, and dyslipidemia, are established as risk factors for CHD reported in many studies. The number of aging patients with CHD is associated with increased morbidity and mortality but also medical treatment, stent placement, and coronary artery bypass graft (22). In the EMPA-REG OUTCOME trial, empagliflozin reduced the risk of MACEs in patients with DM and atherosclerotic cardiovascular disease (23). In addition to placing older adults at increased risk for CHD, dyslipidemia may cause a rapid aggravation of the long-term prognosis, such as frequent premature death, multiple ischemic recurrences, and multivessel disease, in younger adults who develop CHD early in life (24). Familial hypercholesterolemia also increased mortality and increased risk of recurrent AMI after the first AMI event (25). Thus, the US and European guidelines recommend for high-risk patients, such as those with a recent MI, to aggressively lower low-density lipoprotein cholesterol levels (26). From the above mentioned, therefore, we have every reason to believe that the same risk factors should be paid more attention to control in both primary and secondary prevention of CHD.

Patients with AF with a CHA_2_DS_2_-VASc score of 1 or more for men and 2 or more for women are likely to benefit from antithrombotic therapy. However, the female sex is an age-dependent stroke risk modifier rather than a risk factor *per se* (27). In our study, logistic regression analysis confirmed that the male sex was an independent risk factor for MI recurrence but not the female sex; therefore, 2 points were assigned to the male sex instead of the female sex in the MCHA_2_DS_2_-VASc score. One observational study indicates that rates of recurrent MI, recurrent CHD events, and mortality in the first year after MI were higher among men than women (28). However, another study found that women experienced a large excess risk of recurrence after MI, independently of clinical characteristics (29).

Possible reasons for sex differences in the predictive value of MCHA_2_DS_2_-VASc score for recurrent events include the complexities of the interactions of risk factors and the effects of certain risk factors stratified by sex are incompletely captured by available data. In addition, the number of male patients with MI in our study was significantly larger than that of women. In addition, low-risk factors often result in a low hospitalization rate. We confirmed that the value of MCHA_2_DS_2_-VASc would be more discriminatory for “truly low-risk” female patients with a rising population.

### Limitations

Some limitations should be considered in our study. First, given the retrospective nature of our study, data are likely to have a certain extent of bias. Hospitalization for patients with MI with mild symptoms and few cardiovascular risk factors is much lower. Thus, the incidence of recurrent MI might be overestimated. Our analysis was conducted with the patients consecutively recruited from three large medical centers, which could partly reduce this selection bias. We also acknowledge that our data were based on electronic medical records from Hospital Information System that are not created for research purposes. However, since the system is routinely used in clinical practice, the data are sufficiently accurate, complete, and full for this study purpose. The degrees of underreporting and misdiagnosis rate for risk factors in hospital registers are often low, which leads to the data with high validity. In addition, the in-hospital duration and observation periods are not fully described, quantified, and compared. However, since the analysis was the occurrence of in-hospital AMI, which occurred less than 28 days, the results of our study were irrelevant to these missing data. Our retrospective results should be confirmed by prospective cohort studies. Second, recurrent MI risk is a continuum. The MCHA_2_DS_2_-VASc score has a fair predictive value of artificially categorizing patients with MI into low and high-risk strata, only with a greater focus on the identification of “high-risk” patients. To improve identifying “truly low-risk” patients, it is necessary to be more inclusive of common recurrence risk factors as part of the MCHA_2_DS_2_-VASc score. Third, our analyses were based on data from inpatients with MI, and the results may not be entirely generalizable to other settings where comorbidity is less prevalent. It seems to increase the relative risk estimates in the analysis of provoked MI recurrence. Similarly, since our study is limited to Chinese individuals, it may be difficult to extrapolate our findings to other populations. Fourth, given that the MCHA_2_DS_2_-VASc score should be applicable to most patients with MI for most of the time and situations in everyday clinical practice, our study did not cover treatment and laboratory data. Therefore, it is not possible to exclude the effects of the paucity of data on the risk of recurrence.

## Conclusion

CHA_2_DS_2_-VASc and MCHA_2_DS_2_-VASc scores are both validated in recurrent MI prediction. However, MCHA_2_DS_2_-VASc has a better predictive value than CHA_2_DS_2_-VASc in overall and male patients with MI and, importantly, should be considered as being a similar predictive value to CHA_2_DS_2_-VASc in female patients. MCHA_2_DS_2_-VASc shifts toward a greater focus on the identification of high-risk patients with MI, and as good as CHA_2_DS_2_-VASc focus on identifying low-risk patients in women. The next step is to expand data collection for risk factors that are unique to and more common in women than men and to improve cardiovascular prevention models for women.

## Data availability statement

The raw data supporting the conclusions of this article will be made available by the authors, without undue reservation.

## Ethics statement

The studies involving human participants were reviewed and approved by the Ethics Committee of the First Affiliated Hospital of Nanjing Medical University. Written informed consent for participation was not required for this study in accordance with the national legislation and the institutional requirements.

## Author contributions

XL conceived and designed the study and supervised all the work. HP collected the data, planned the analyses, and drafted the manuscript. XZ and IC performed all calculations and interpreted the data. HZ and YZ designed the case report forms. SL supervised the statistical analyses. All authors contributed intellectually to the manuscript, reviewed drafts, and accepted the final draft.
